# The Histone H3 K4me3, K27me3, and K27ac Genome-Wide Distributions Are Differently Influenced by Sex in Brain Cortexes and Gastrocnemius of the Alzheimer’s Disease PSAPP Mouse Model

**DOI:** 10.3390/epigenomes5040026

**Published:** 2021-11-25

**Authors:** Francesca Casciaro, Giuseppe Persico, Martina Rusin, Stefano Amatori, Claire Montgomery, Jennifer R. Rutkowsky, Jon J. Ramsey, Gino Cortopassi, Mirco Fanelli, Marco Giorgio

**Affiliations:** 1 Department of Biomedical Sciences, University of Padua, Via Ugo Bassi 58/B, 35131 Padova, Italy; 2 Department of Experimental Oncology, IRCCS—European Institute of Oncology, Via Adamello 16, 20139 Milano, Italy; giuseppe.persico@ieo.it (G.P.); martina.rusin@ieo.it (M.R.); 3 Molecular Pathology Laboratory “PaoLa”, Department of Biomolecular Sciences, University of Urbino Carlo Bo, Via Arco d’Augusto 2, 61032 Fano (PU), Italy; stefano.amatori@uniurb.it (S.A.); mirco.fanelli@uniurb.it (M.F.); 4 School of Veterinary Medicine, University of California, Davis, CA 95616, USA; cbmontgomery@ucdavis.edu (C.M.); jrutkowsky@ucdavis.edu (J.R.R.); jjramsey@ucdavis.edu (J.J.R.); gcortopassi@ucdavis.edu (G.C.)

**Keywords:** Alzheimer’s disease, sex differences, PSAPP mice, histone marks

## Abstract

Background: Women represent the majority of Alzheimer’s disease patients and show typical symptoms. Genetic, hormonal, and behavioral mechanisms have been proposed to explain sex differences in dementia prevalence. However, whether sex differences exist in the epigenetic landscape of neuronal tissue during the progression of the disease is still unknown. Methods: To investigate the differences of histone H3 modifications involved in transcription, we determined the genome-wide profiles of H3K4me3, H3K27ac, and H3K27me3 in brain cortexes of an Alzheimer mouse model (PSAPP). Gastrocnemius muscles were also tested since they are known to be different in the two sexes and are affected during the disease progression. Results: Correlation analysis distinguished the samples based on sex for H3K4me3 and H3K27me3 but not for H3K27ac. The analysis of transcription starting sites (TSS) signal distribution, and analysis of bounding sites revealed that gastrocnemius is more influenced than brain by sex for the three histone modifications considered, exception made for H3K27me3 distribution on the X chromosome which showed sex-related differences in promoters belonging to behavior and cellular or neuronal spheres in mice cortexes. Conclusions: H3K4me3, H3K27ac, and H3K27me3 signals are slightly affected by sex in brain, with the exception of H3K27me3, while a higher number of differences can be found in gastrocnemius.

## 1. Introduction

According to the last World Alzheimer Report and most USA and EU statements, there are more than 55 million people living with dementia worldwide in 2021, with two-thirds of the clinically diagnosed cases in women [1,2,3]. The most common type of dementia is Alzheimer’s disease (AD) whose prevalence is found to be higher in females than males according to several studies [3,4]. Women also show a wider spectrum of AD symptoms [5] and a faster consequent cognitive decline [6].

AD is characterized by extracellular deposition of the amyloid β (Aβ) peptide, a product of amyloid precursor protein (APP) processing, and by intraneuronal neurofibrillary tangles of hyperphosphorylated tau protein. In non-pathological conditions, APP is cleaved by α-secretase and consequently by the γ-secretases (PSEN), while in the amyloidogenic pathway, the β-secretase enzyme cleaves APP to produce a soluble fragment which is then cleaved by γ-secretase to release the Aβ peptide [7]. Consistently, mice expressing the mutated human γ-secretases 1 and the mutant chimeric mouse/human mutant APP (Mo/HuAPP695swe) (PSAPP mice) accumulate Aβ toxic plaques in the brain cortex and develop behavioral hallmarks of AD patients [8].

AD is a complex disease with a multiorgan involvement. Slow gait speed and decreased grip strength are associated with cognitive decline [9], and muscle is known to be one of the tissues with the most sex-differentially expressed genes in humans [10].

In a 3xTg-AD mouse model, which contains three gene mutations, namely APPSwe, PS1M146V, and tauP301L, it has been reported that disfunctions in skeletal muscle occur at different levels. For example, they showed that skeletal muscle functionality is already affected in 3-month-old 3xTg-AD mice with an age-dependent accumulation of amyloid-β1-40 peptide [11]. Furthermore, in a double transgenic mouse model expressing a chimeric mouse/human amyloid precursor protein (APP) with the Swedish mutation (APPswe) and a mutant human presenilin 1 (PS1) with the delta E9 (PS1ΔE9), it has been shown that skeletal muscle cells have a significantly decreased maximal mitochondrial oxygen consumption capacity compared to non-transgenic, age-matched mice, with similar deficits to those previously described in brain [12].

In the last years, multiple studies have shown the involvement of epigenetic regulation in the progression of AD. For example, it was found that some cytosines, particularly those at -207 to approximately -182 in the promoter region of the APP gene, are mostly methylated and their demethylation with age may lead to Aβ deposition in the aged brain [13]. Moreover, different studies in mouse models of AD have shown an involvement of histone deacetylases in speeding up the progression of the disease [13].

Emerging evidence from animals and humans suggests that epigenetic mechanisms or environmental factors are likely to also play a role in the different incidence and progression of the disease in the two sexes [14].

A good example of sex-specific epigenetic regulation is X chromosome inactivation in females, a process which occurs largely due to a combination of DNA methylation and histone modifications [15].

Recently, it has been proposed that histone modifications may be involved in the different responses to stress between males and females [16]. For example, Ramzan et al. have shown that the expression of histone variant H2A.Z has context-specific effects on the regulation of fear memory and related disorders, so higher levels of H2A.Z in female mice may represent a risk factor for PTSD and associated increased pain sensitivity [17]. Furthermore, analysis of the bed nucleus of the stria terminalis and preoptic area in adult male and female mice revealed 248 regions differently enriched in H3K4me3 in the two groups [18].

Here, we explore the landscape of three well characterized transcription-associated histone modifications, both in brain cortexes and gastrocnemius muscle in a PSAPP mouse model of AD. In particular, H3K4me3 and HK27ac are associated with gene activation, while H3K27me3 is correlated with repression and especially with the inactivation of one copy of the X chromosome in females [19]. Multiple studies correlate alterations in the signal of those histone marks with the aging process. For example, high levels of H3K27me3 were observed in the brain of mice with an accelerated aging phenotype [20], while global histone acetylation was found regulated in aged mice brain [21]. Alterations in H3K4me3 signal was observed in different aged tissues of mouse models [22,23]. In humans, H3K4me3 distribution in prefrontal neurons from 11 individuals resulted to decrease in 600 loci in early life and to increase in other 100 loci of aged adults [24].

Chromatin immunoprecipitations, followed by Illumina sequencing, were conducted for these modifications and bioinformatics analyses results are here presented.

## 2. Materials and Methods

### 2.1. Mice

Five female and five male PSAPP mice were generated at the mouse facility of the University of California (Davis, CA, USA) and housed in polycarbonate cages on racks in a room with controlled temperature (22–24 °C) and humidity (40–60%). Mice were individually housed in a HEPA filtered room maintained on a 12-hr light–dark cycle. Health checks were conducted on all mice at least once a day. Sentinel mice were housed in the same room and exposed to bedding from the study mice on a weekly basis. Health screens were completed on sentinel mice every three months. Serology tests included MHV, Sendai, PVM, MPV, MVM, M.pul and arth, TMEV (GDVII), Ectro, EDIM, MAD1 and 2, LCM, Reo-3, and MNV. All tests were negative throughout the study. All animal protocols were approved by the UC Davis Institutional Animal Care and Use Committee and were in accordance with the NIH guidelines for the Care and Use of Laboratory Animals.

Mice were multi-housed and provided ad libitum access to a chow diet LabDiet 5001 (LabDiet, Saint Louis, MO, USA) prior to the start of the study. At 6 months of age, mice were singly housed and placed on a modified AIN-93 diet (Table S12). Food intake was set at 11.2 kcal/day, which was fed ~1 h prior to lights out, and water was provided ad lib.

Brain cortexes and gastroectonemius muscles were collected from sacrificed mice following standard procedures [25,26].

### 2.2. ChIPSeq

Brain cortexes and gastrocnemius muscle chromatins were obtained from five male (*n* = 4 for male cortex) and five female PSAPP-mice sacrificed at the age of 13 months when these animals begin to show symptoms of cognitive decline and immediately snap frozen in liquid nitrogen.

Chromatin extraction was performed on 10-mg tissues following the chromatin immunoprecipitation (ChIP) standard procedure [27].

Extracted chromatin was immunoselected with anti-H3K4me3 (#39159, Lot 22119006, Active Motif, Carlsbad, CA, USA), anti-H3K27ac (ab4729, Lot. GR3231887-1; Abcam, Cambridge, UK) and H3K27me3 (07-449, Lot. 3091919; Millipore, Temecula, CA, USA) antibodies following the procedure already described without limited reversal of crosslinking (LRC) step [28].

The bound fractions were de-crosslinked, and purified DNA was used for library preparation. Libraries were then sequenced in 51 bp paired-end read mode on a NovaSeq 2000 sequencer (Illumina, San Diego, CA, USA).

Sample c05 was excluded from the analysis due to problems during the preparation.

### 2.3. Computational Pipeline

Reads were aligned to mm10 using “bwa” (v0.7.17), a software package for mapping low-divergent sequences against a large reference genome [29]. Unmapped reads, reads with a mapping quality (MAPQ) value smaller than 1, duplicate reads, and those that mapped outside of chr 1–19 and Chr X were removed using SAMtools. Resulting alignment reads (stored in a standard BAM format, which is the compressed binary version of a SAM file that is used to represent aligned sequences up to 128 Mb) were converted into a bedpe format (browser extensible data paired-end format, which helps to concisely describe disjoint genome features, such as structural variations or paired-end sequence alignments) using the “bamTobed” script of bedtools (v2.30.0) [30]. Peak detection was performed with epic2 software using the following parameters: fragment size = 200; window size = 200; g = 2 for H3K4me3 and H3K27ac; g = 3 for H3K27me3; FDR < 0.05; and e = 100 [31]. Bedtools was also used to merge the peak files of each group prior to annotating them.

Differential binding analysis was performed by the Diffbind R package (v3.2.6) and differentially bound sites were identified among different conditions. Stringency in the analysis was obtained by creating a consensus dataset for each condition, including peaks that were present in at least three samples of the considered group. Only different bound (DB) sites with an FDR (false discovery rate) of <0.1 were considered [32].

The ChIPseeker R package (v1.28.3) was applied to annotate peak files and DB sites using the curated RefSeq set version 130306 [33].

Pathway analysis was conducted using the ingenuity pathway analysis (IPA) software from QIAGEN (version September 2021). Pathways with an absolute z-score of >2 were considered significant.

For some analyses, BAM files of replicates from each group were merged using BAMtools and indexed using SAMtools. Merged bam files were then used to generate a bigwig (a file format for display of dense, continuous data in a genome browser track) using deepTools bamCoverage (v3.5.1) with a bin size of 10 bp (size of the bins, in bases), bins per million (BPM) mapped reads, normalization (ChrX was ignored for normalization), and reads extended to 200 bp.

The signal around the TSSs was calculated for 23,359 genes. The signal, calculated using deepTools computeMatrix, was reported as a mean signal in bins of 10 bp, with a range of ±3 kB around the TSSs. Missing data were treated as zero. The output was then plotted using plotHeatmap and plotProfile (deepTools) [34]. 

## 3. Results

### 3.1. H3K4 and H3K27 me3 Analysis on X Chromosome Distinguishes Males and Females

We first checked the quality of sequencing results and no significant variability in the number of reads related to sex was revealed (Figure S1).

**Figure 1:**
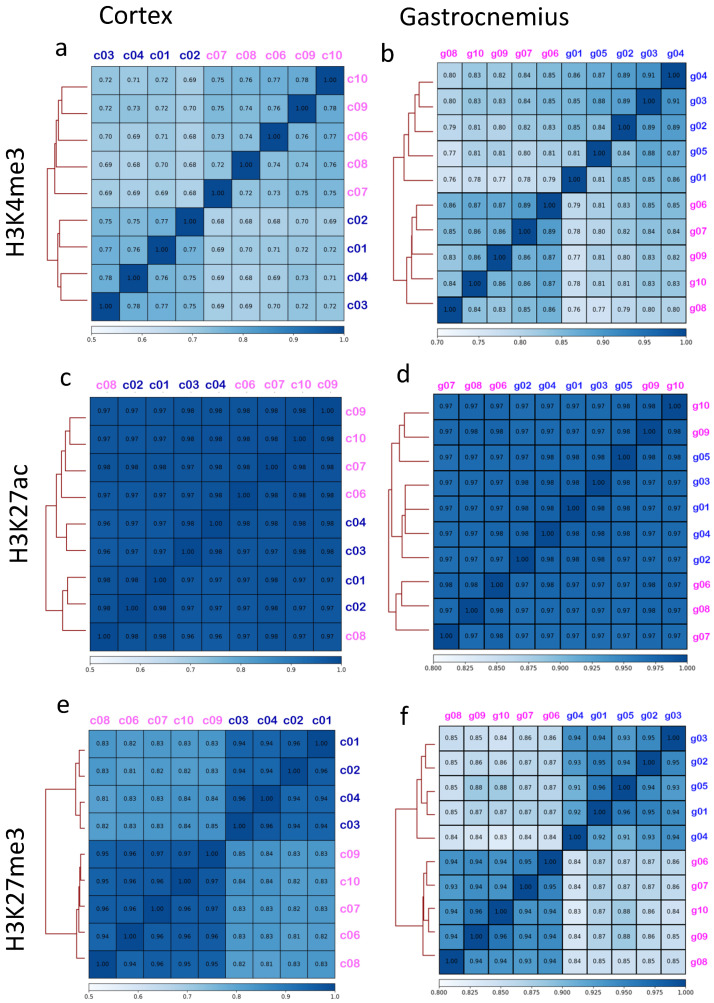
Spearman correlation including autosomes and X chromosome. Spearman correlation heatmap including chromosomes 1-19 and X for H3K4me3 (**a**), H3K27ac (**c**), and H3K27me3 (**e**) from cortexes are represented in the graphs on the left side. Corresponding graphs from gastrocnemius, for H3K4me3 (**b**), H3K27ac (**d**), and H3K27me3 (**f**), are reported on the right side. Male IDs are indicated in blue, female IDs are in pink. The color within each cell represents the Spearman coefficient which ranges from 0.68 to 1 (maximal correlation).

To estimate differences in the distribution of the analyzed histone’s marks between females and males, we performed a correlation analysis, including all the aligned reads in autosomes (chromosomes, chr 1-19) and X. According to Spearman correlation analysis, males and females formed distinguishable clusters for H3K4me3 (Figure 1a,b) and for H3K27me3 (Figure 1e,f), which is known to be involved in the X chromosome inactivation [35]. In contrast, H3K27ac showed higher correlation coefficients (above 0.9), indicating a greater similarity among all the analyzed samples (Figure 1c,d). As expected, the H3K4me3 and H3K27me3 sex-related correlation was lost if only autosome chromosomes are considered, underlining the contribution of the X chromosome in generating this phenomenon (Figure S2).

### 3.2. Brain Cortex Shows a Signal around the Transcription Starting Sites (TSS) That Is More Homogenous Than Gastrocnemius

**Figure 2:**
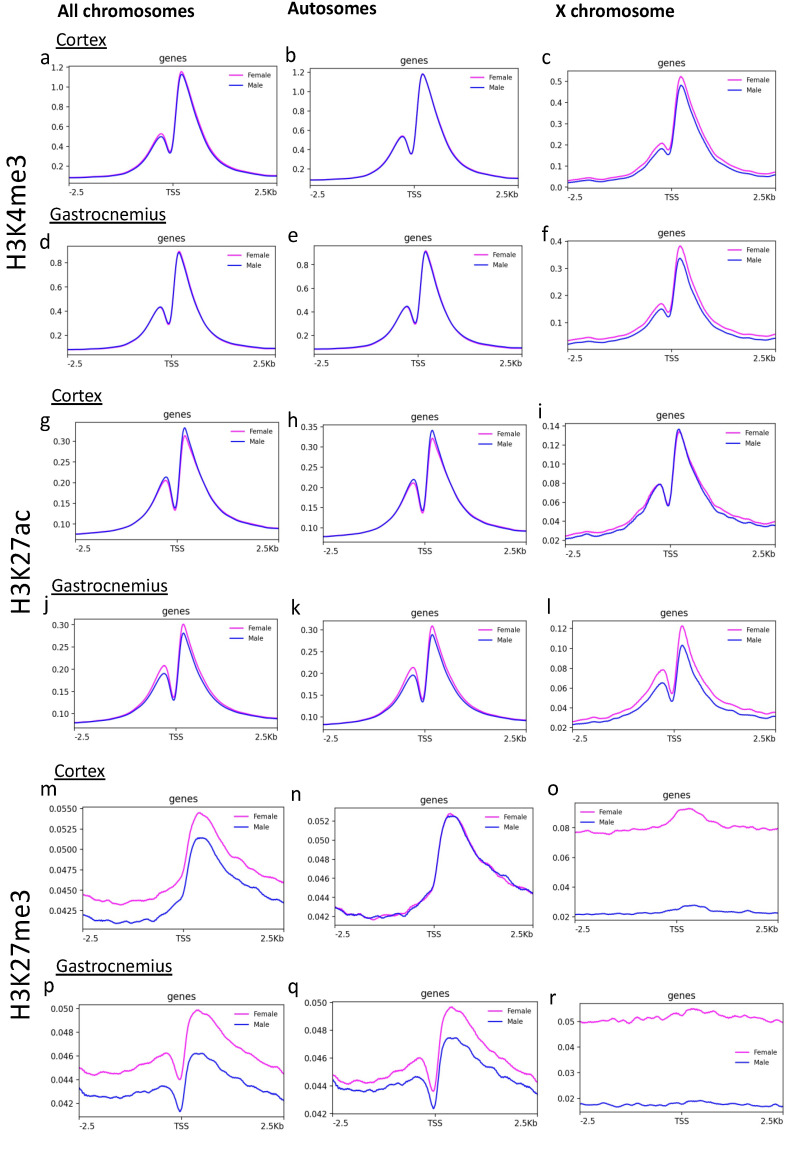
Signal around TSS of H3K4me3, H3K27ac, and H3K27me3 in cortex and gastrocnemius tissues. H3K4me3 (**a**–**f**), H3K27ac (**g**–**l**), and H3K27me3 (**m**–**r**) average density signal around TSS (±2.5 kb) is plotted for cortex and gastrocnemius as indicated, for chromosomes 1–19 and X on the left, only autosomes in the middle and only X on the right. Males are indicated in blue, females in pink.

Since histone modifications play a critical role in transcription control, we investigated the signal around (−2.5 kb + 2.5 kb) the TSS present in all the chromosomes (*n* = 23,359 sites). The comparison of the average signal from all the TSS revealed a substantial difference between the two sexes only for H3K27me3 (Figure 2m,p), and not for H3K4me3 and H3K27ac (Figure 2a,d,g,j).

Focusing on the analysis of the average signals on TSS separating autosomes and the X chromosome, the intensity of the H3K27me3 signal on the X chromosome scored almost double in females than males in both cortex and gastrocnemius (Figure 2o,r). In the gastrocnemius, a slight difference in the H3K27me3 signal on TSS was also found if considering only the autosomes. Differences in the H3K27ac signal on TSS also appeared on the X chromosome of gastrocnemius.

These results indicate that sex differences of histone modifications distribution in the cortex of PSAPP mice occur to a lesser extent in comparison to the gastrocnemius, whose fiber composition and gene expression is influenced by sex [36,37].

### 3.3. Binding Sites Are Differently Affected by Sex in Cortex and Gastrocnemius

**Figure 3:**
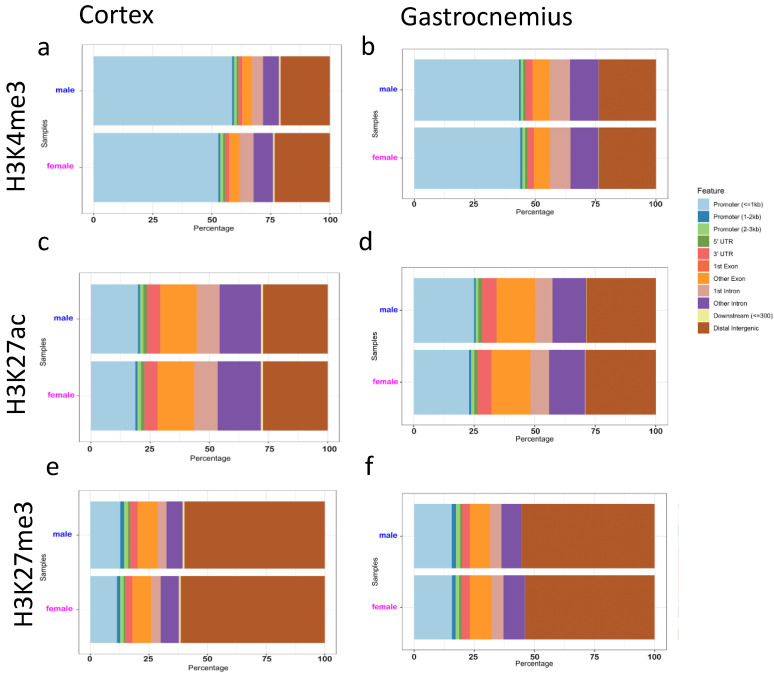
Feature distribution of called peaks for H3K4me3, H3K27ac, and H3K27me3 in cortex and gastrocnemius tissues. Feature distribution of the identified peaks for H3K4me3 (**a**), H3K27ac (**c**), and H3K27me3 (**e**) in cortexes are reported on the left. Feature distribution of H3K4me3 (**b**), H3K27ac (**d**), and H3K27me3 (**f**) peaks found in the gastrocnemius are reported in the plots on the right. Males are in blue, females in pink. The color code indicating each genomic feature is shown.

The position of the marked H3 histones was investigated genome-wide using the epic2 tool to identify enriched peaks. Overall, the number of peaks detected in the two sexes appeared to be comparable (Figure S3), except for the H3K4me3 in the female cortex, whose number was higher in three out of five samples. The analysis of peak distribution within the different genes features revealed that most H3K4me3 peaks were found in promoters (Figure 3a,b), although distal intergenic regions were more represented in female cortexes (Figure 3a). H3K27ac peaks were more uniformly distributed among the different features (Figure 3c,d), whereas the majority of the H3K27me3-enriched regions were identified in distal intergenic areas (Figure 3e,f).

Then, we identified the sites that showed different intensity of histone marks signals between the sexes (Diffbind analysis). To avoid an excess of false positive results due to the unspecific binding of the antibody during the ChIP, we generated a consensus dataset of peaks for each group (Table S1), as reported in the methods section, and we separately analyzed autosomes and X chromosome.

**Figure 4:**
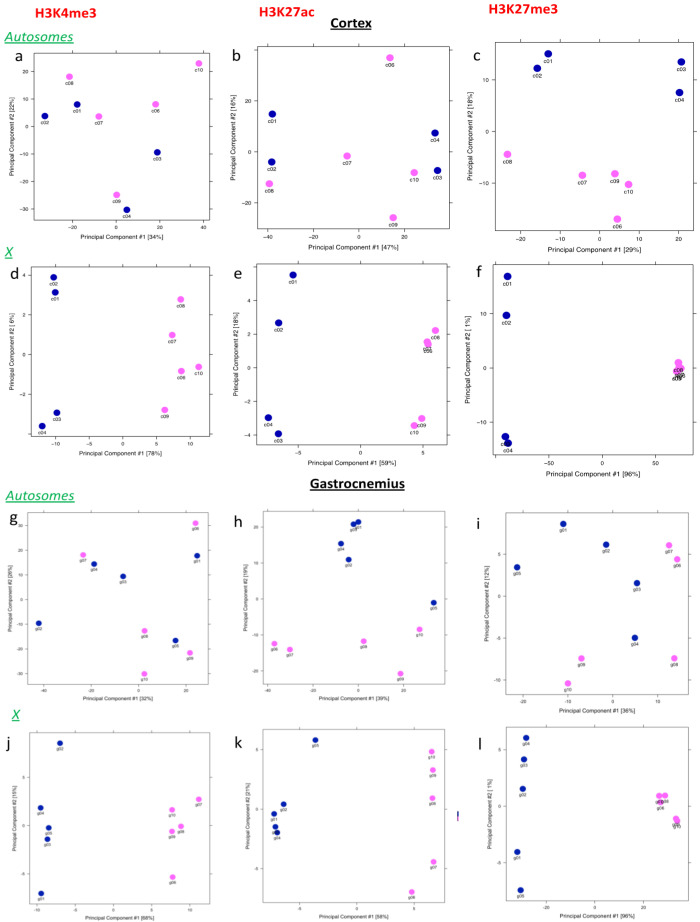
Principal component analysis of Diffbind consensus peak sets. Principal component analysis of the signals from regions selected after differential binding analysis are graphed. The left column reports information on H3K4me3, the middle one reports on H3K27ac, while H327me3 is analyzed on the right part of the figure.

To investigate the heterogeneity in these sets of peaks among samples, principal component analyses (PCA) were performed. Results revealed that females and males separate only if considering the X chromosome (Figure 4d–f,j–l)**,** with the principal component 1 showing the maximum variance for H3K27me3 (96% for both tissues) (Figure 4f,l) and the minimum for H3K27ac (around 55/59%) (Figure 4e,k). PCA for the H3K4me3 X chromosome resulted in a more variable component 1 between the two sexes, with a higher variance shown by cortexes (78%) (Figure 4d,j). Taking in account autosomes, male and female H3K4me3 and H3K27ac differentially marked peaks appeared and merged for both tissues (Figure 4a,b,g,h).

The figure can be divided in two parts: the upper part presents the results related to the cortex for autosomes (a–c) and the X chromosome (d–f) for all the histone marks analyzed, while the lower part shows the principal component analyses of autosomal (g–i) and X (j–l) chromosomes in muscle.

Considering autosomes, the Diffbinding analysis between males and females identified only two sites for H3K4Me3 and H3K27ac and five for H3K27me3 in the cortex, whereas 139 H3K4me3, 206 H3K27me3, and 781 H3K27ac sites were discovered in the gastrocnemius (Table 1).

Regarding the X chromosome, Diffbind identified 65 H3K4me3, 56 H3K27ac, and 2782 H3K27me3 sex differential sites in the cortex, while 102 H3K4me3, 69 H3K27ac, and H3K27me3 sex differential sites in the gastrocnemius (Table 1). The investigation of the location of these sites (Tables S2–S7) revealed that a larger number of sex differential promoters involves the H3K27me3 in the X chromosome and the H3K27ac in the autosomes.

### 3.4. Genes Involved in Cognitive Functions Show Different H3K27me3 Signal between Sex

To disclose the function of the genes, whose promoters were differently histone-marked by sex, ingenuity pathway analysis (IPA) was performed on the different lists of the identified genes. IPA is a web-based software application for the analysis, integration, and interpretation of data derived from OMICS experiments, and it is able not only to categorize genes in pathways and biological functions, but it can give an idea of the direction of the regulation (expressed by a positive or negative z-score) [38]. Due to the low number of genes differentially marked between the sexes in the cases of H3K4me3 and H3K27ac, few terms for biological processes were recognized (Tables S8–S11). For IPA analysis on the genes whose promoters showed different sex-related H3K27me3 peaks (*n* = 488), the majority of these genes were classified as involved in processes linked to neuronal cell functions and several to behavioral functions (Table 2). Notably, among the genes assigned with these pathways, eight (HSD17B10, GATA1, HTR2C, OGT, AGTR2, CYBB, GRIA3, MAOA) were reported to be associated to AD according to the GeneCards database (https://www.genecards.org/, accessed on 27 September 2021) with a relevance score superior to two [39,40,41,42,43,44,45,46].

In the gastrocnemius, the sex-dependent H3K27ac-marked promoters (*n* = 123) showed higher signals in females on genes related to inflammation and lower signals on those related to apoptosis (Table S10). Genes assigned with these terms are the brain-derived neurotrophic factor (BDNF), the histamine N-methyltransferase (HNMT), and the insulin receptor factor 1 (IRS1), which are all associated with AD (with a relevance score of 14.34, 76, and 3.65, respectively) [47,48,49].

Finally, the cortex shares with gastrocnemius sex differences in H3K27me3 promoters of genes involved in cytoskeleton organization (Table 2 and Table 3).

## 4. Discussion

As observed in patients and in animal models of AD, the disease progresses differently in the two sexes. The brains of PSAPP female mice accumulate significantly more amyloid plaques than male mice [50] and show more severe angiopathy and inflammation [51] from 6 months of age. Consistently, PSAPP female mice show lower cognitive abilities than PSAPP male mice [52]. Indeed, neurodegeneration and cognitive decline are worse among females from different models of mice with dementia [53]. Because of this difference in susceptibility to the disease, the question arises as to whether epigenetic signal may be involved in the different prevalence of the diseases between sexes.

In the present study, we determined the H3K4me3, H3K27ac, and H3K27me3 genome-wide profiles of cortexes from 13-month-old PSAPP male and female mice. The comparison of the global distribution of H3K4 and H3K27me3 signals revealed differences in the X chromosome capable to distinguish males and females, whereas the H3K27ac distribution was more homogenous between sexes. As expected, this sex-dependent regulation was particularly evident for the H3K27me3 signal around the TSS of genes localized on the X chromosome, since the inactivation process of genome portions of one of the two X chromosomes present in females.

Searching for differently bound sites also confirmed that the epigenetic landscape for the three considered histone modifications in the PSAPP mice is less influenced by sex in the brain than in muscle, whose transcriptomic profile is known to be particularly different between sexes [54]. However, a significantly different level of H3K27me3 on the X chromosome was observed in the cortex of male and female PSAPP mice. Interestingly, several of these X chromosome sites are involved in the regulation of neuronal functions, such as spatial learning, cellular organization, and development, and some of them are reported to be associated with AD [39,40,41,42,43,44,45,46].

Autosomes and the sex chromosomes differ in their evolutionary origins and in their involvement in cognitive functions. Despite many similarities between females and males, sex differences are present in learning and memory [55]. Notably, in humans, 3.75% of all genes are located on the X chromosome [56] and almost one third of these genes are involved in cognitive functions [57]. In females, one of the copies of the X chromosome is silenced. This process of X-chromosome inactivation evolved as a mechanism to regulate gene dosage. However, it does not affect all genes equally and those genes that are differently regulated, particularly under stress conditions, such as the appearance of amyloid plaques in the brain, may impact on the progression of neuronal disease.

Histone modifications are reported to be involved in the genome remodeling during learning and memory [58]. In particular, an increase in H3K4me3 in the mouse hippocampus has been related to long-term memory [59]. Numerous studies indicate that hippocampus-dependent memory and synaptic plasticity may rely on histone acetyltransferases and histone deacetylases activity [16]. However, similar to histone acetylation, the majority of studies on histone methylation have exclusively used males [16]. Thus, the impact of histone methylation in mediating sex- dependent memory processes are not well understood. Some evidence suggests that the activity of histone methyltransferases and demethylases may be influenced by sex. For example, the histone demethylase KDM5C and UTX are coded by X-linked genes and escape X-inactivation in females, and may mediate sex differences in brain development, memory, and behavior [60,61].

In conclusion, this study reveals that the chromatin of brain cortex from PSAPP mice shows a sex-dependent signature of histone modifications distinct from other tissues, such as the gastrocnemius. Epigenetic signals have been suggested to be involved in sex-dependent cognitive decline even if, so far, no epigenomes of male and female cortexes of AD models are available. The results presented here show that important sex differences exist in the distribution of histone modifications in transcription control regions of several genes involved in neuronal functions that may be involved in the cognitive decline in AD patients.

## Figures and Tables

**Table 1:** Differentially bound sites of H3K4me3, H3K27ac, and H3K27me3 in cortex and gastrocnemius tissues. The number of the differentially bound sites (FDR < 0.1) found in each comparison is reported.

Histone Modification	Chromosomes	N° of db Sites in Cortex	N° db Sites in Gastrocnemius
	Autosomes	2	139
H3K4me3	X chromosome	65	102
	Autosomes	2	781
H3K27ac	X chromosome	56	69
H3K27me3	Autosomes	5	206
	X chromosome	2783	855

**Table 2:** Biological signatures associated to H3K27me3-regulated promoters on the X chromosome in cortex. Results of an ingenuity pathway analysis for promoters differentially marked by H3K27me3 in X chromosome from PSAPP cortex are reported. Only terms with an activation z-score above |2| are considered. All the results are presented as increased/decreased in females with respect to males.

Cortex Diseases or Functions (H3K27me3 X)	*p*-Value	Predicted Activation State	Activation Z-Score	Molecules	N° of Molecules
Spatial learning	2.70 × 10^−^^5^	Increased	2.646	AP1S2,ARHGEF9,CYBB,DLG3,Gprasp2,GRIPAP1,HTR2C, KDM5C, MECP2, OPHN1,PHF8,SLC6A8,UBE2A, ZDHHC9	14
Growth of neurites	4.98 × 10^−3^	Increased	2.138	AR,ARX,CCDC120,CDKL5,DCX,EFNB1,ELK1,FRMD7, GJB1, MAO, MID1,OGT,PLXNA3,RAB33A,SLC25A5, SNX12,SYN1,TLR7,TRPC5	19
Organization of cytoskeleton	1.17 × 10^−^^7^	Increased	2.074	AGTR2,AMOT,AR,ARHGAP4,ARHGAP6,ARHGEF9, ATP7A, BRWD3,CAPN6,CDK16,CDKL5,CETN2,CUL4B, CXCR3,CYBB, DCX,DGKK,DLG3,DOCK11,EFNB1,ELK1, F8A1 (includes others),FGD1,FLNA,FRMD7,GATA1,GDI1,GJB1,GPM6B, Gprasp2,HDAC6,HDAC8,HPRT1, IL1RAPL1,KDM5C,MAOA, MECP2,MID1,MID1IP1, mir-384,MPP1,MTM1,NR0B1,OFD1, OGT,OPHN1,PAK3, PCYT1B,PLS3,PLXNA3,PLXNB3,POF1B, PQBP1,RAB33A, RPGR,RPS6KA3,SH3KBP1,SHROOM2, SHROOM4, SLITRK2,SYN1,TLR7,Tmsb4x (includes others), TRPC5, USP9X	65
Organization of cytoplasm	8.76 × 10^−7^	Increased	2.074	AGTR2,AMOT,AR,ARHGAP4,ARHGAP6,ARHGEF9, ATP7A, BRWD3,CAPN6,CDK16,CDKL5,CETN2,CUL4B,CXCR3,CYBB, DCX,DGKK,DLG3,DOCK11,EFNB1,ELK1,F8A1(includes others),FGD1,FLNA,FRMD7,GATA1,GDI1,GJB1,GPM6B, Gprasp2,HCFC1,HDAC6,HDAC8,HPRT1,HSD17B10, IL1RAPL1,KDM5C,MAOA,MECP2,MID1,MID1IP1,mir-384, MPP1,MTM1,NR0B1,OFD1,OGT,OPHN1,PAK3,PCYT1B,PLS3,PLXNA3,PLXNB3,POF1B,PQBP1,RAB33A,RPGR,RPS6KA3, SH3KBP1,SHROOM2, SHROOM4, SLITRK, SYN1,TLR7, Tmsb4x (includes others),TRPC5,USP9X	67
Tremor	1.14 × 10^−3^	Decreased	−2.011	ARAF,CA5B,GABRQ,GJB1,GPM6B,GRIA3,IKBKG,MECP2, PLP1, TIMP1	10
Differentiation of Th2 cells	1.09 × 10^−2^	Decreased	−2.236	FOXP3,GATA1,IL13RA2,let-7,TLR7	5
Movement Disorders	2.29 × 10^−3^	Decreased	−2.242	ABCB7,AIFM1,AMER1,AP1S2,AR,ARAF,ARHGEF9,ARMCX2, AR, ATP6AP2,BCAP31,CA5B,CDKL5,CETN2,CXCR3,F8A1 (includes others),GABRQ,GJB1,GPM6B,GRIA3,GRPR,HPRT1, HTR2C,IDS,IGSF1,IKBKG,KCND1,MAOA,MECP2,OGT,PDK3, PGK1,PGRMC1,PLP1,PRKX,PTCHD1,RGN,RS1,SRPX,SRPX2, SYN1,SYTL4,TIMP1,Tmsb4x (includes others),XIAP	45
Motor dysfunction or movement disorder	1.72 × 10^−3^	Decreased	−2.666	ABCB7,AIFM1,AMER1,AP1S2,AR,ARAF,ARHGEF9,ARMCX2, AR,ATP6AP2,BCAP31,CA5B,CDKL5,CETN2,CXCR3,F8A1 (includes others),GABRQ,GJB1,GPM6B,GRIA3,GRPR,HPRT1, HTR2C,IDS,IGSF1,IKBKG,KCND1,MAOA,MECP2,MTM1, OGT, PDK3,PGK1,PGRMC1,PLP1,PRKX,PTCHD1,RGN,RS1, SRPX,SRPX2,SYN1, SYTL4,TIMP1,Tmsb4x (includes others), XIAP	46

**Table 3:** Biological signatures associated to H3K27me3-regulated promoters on gastrocnemius X chromosome. Results of an ingenuity pathway analysis for promoters differentially marked by H3K27me3 in X chromosome from PSAPP gastrocnemius are reported. Only terms with an activation z-score above |2| are taken in consideration. All the results are presented as regulated (increased/decreased) in females with respect to males.

Gastrocnemius Diseases or Functions (H3K27me3 X)	*p*-Value	Predicted Activation State	Activation Z-Score	Molecules	N° of Molecules
Organization of actin cytoskeleton	27.23 × 10^−3^	Increased	2.000	ARHGAP4,EFNB1,FGD1,GPM6B,MSN,OPHN1,PAK3,PLS3, Tmsb4x (includes others)	9
Abdominal cancer	1.41 × 10^−5^	Decreased	−2.000	ABCB7,ACE2,AIFM1,AMER1,ARHGAP36,ARHGAP4,ARHGEF9, ARMCX1,ARMCX2,ARMCX3,ARMCX4,ARMCX5,ARX, ASB9, ATP2B3,AVPR2,AWAT2,BCOR,BCORL1,BEX1,BGN, BMX,BRS3, BTK,CA5B,CACNA1F,CCDC160,CD40LG, CDX4,CHRDL1, CLDN2,CNGA2,CNKSR2,COL4A5,DACH2,DCAF12L2,DGAT2L6,DOCK11, DUSP9, EFNB1,EGFL6,ELF4,ERCC6L,FAM155B, FGD1,FOXO4,FOXR2,FRMD7,FRMPD3,GABRA3,GABRQ,GATA1, GDPD2,GJB1,GPC3,GPC4,GPM6,GPR101,GPR143, GPR50,GRIA3, GRPR,GSPT2,GUCY2F,HCFC1,HDAC8, HPRT1,HSD17B1,HTR2C, IDH3G,IGSF1,IL13RA1,IL1RAPL1,IL1RAPL2, IQSEC2,IRAK1, KCND1,KCNE5,KLF8,KLHL15,L1CAM, LANCL3,LHFPL1, LONRF3,MAGEA10, MAGEA11,MAGED1,MAGEE1,MAGEE2, MAP7D2,MBNL3,mir-452,MSN,NAA10,NAP1L2,NEXMIF,NONO, NRK,NYX,OGT,OPHN1,OPN1LW,OTUD6A,PAK3,PCDH11X, PCDH19,PDK3,PDZD4,PHF6,PHKA1,PLAC1,PLS3,PLXNA3, PLXNB3,PNMA3,PNMA5,PRKX,PRPS1,PRPS2,PTCHD1, RAB33A, RAI2,RAP2C,RBM41,RNF128,RPS4Y1,RRAGB,SLC16A2,SLC25A43,SLC38A5,SLC6A8,SLC7A3,SLITRK4,SOWAHD,SOX3,SRPX, STARD8,SYN1,SYTL4,TAB3,TAF1,TBX22,TCEANC,THOC2, TMEM164,TMEM255A,TMEM47,TREX2, TRPC5,TSC22D3,TSPYL2, UPF3B,USP51,UTP14A,VGLL1,YIPF6,ZC4H2, ZCCHC12, ZDHHC9,ZFX,ZIC3,ZMYM3	162

## Data Availability

ChIP-seq data are deposited on GEO repository and accessible with GSE189260 number (https://www.ncbi.nlm.nih.gov/geo (accessed on 4 November 2021)). All the data that support the figures and the other findings are available from the authors upon request.

## Supplementary Materials

The following are available online at https://www.mdpi.com/article/10.3390/epigenomes5040026/s1, Figure S1: Number of aligned reads, Figure S2: Spearman correlation including only autosomes, Figure S3: Number of peaks, Table S1: Number of regions investigated during the differential bounding analyses, Table S2: H3K4me3 differentially bound sites and related annotations in cortex, Table S3: H3K4me3 differentially bound sites and related annotations in gastrocnemius, Table S4: H3K27ac differentially bound sites and related annotations in cortex, Table S5: H3K27ac differentially bound sites and related annotations in gastrocnemius, Table S6: H3K27me3 differentially bound sites and related annotations in cortex, Table S7: H3K27me3 differentially bound sites and related annotations in gastrocnemius, Table S8: Biological signatures associated to H3K4me3-regulated promoters on cortex X chromosome, Table S9: Biological signatures associated to H3K4me3-regulated promoters on gastrocnemius X chromosome, Table S10: Biological signatures associated to H3K27ac-regulated promoters on gastrocnemius autosomes, Table S11: Biological signatures associated to H3K27ac-regulated promoters on gastrocnemius X chromosome, Table S12: Diet composition.
