# Supplementary material for: The Histone H3 K4me3, K27me3, and K27ac Genome-Wide Distributions Are Differently Influenced by Sex in Brain Cortexes and Gastrocnemius of the Alzheimer’s Disease PSAPP Mouse Model
Source: Epigenomes. 2021 Nov 25;5(4):26. doi: 10.3390/epigenomes5040026 (PMC8715457; doi:10.3390/epigenomes5040026)
Supplement: Supplementary file 1 [file epigenomes-05-00026-s001.zip › epigenomes-1442647-SI.pdf]

Supplementary Material

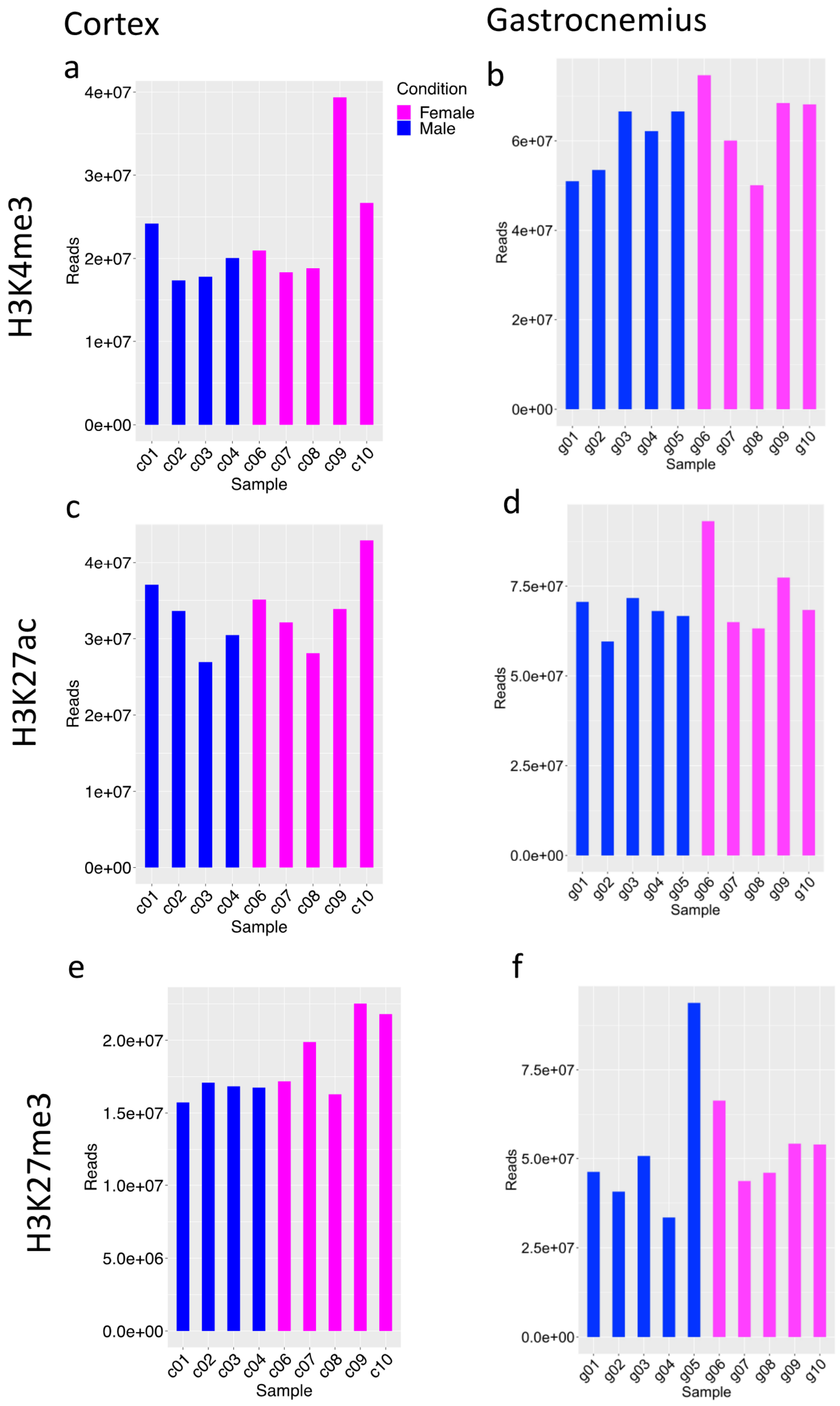

Figure S1. Number of aligned reads. On the left side the number of reads for H3K4me3 **(a)**, H3K27ac **(c)** and H3K27me3 **(e)** in brain is reported. The right part of the figure shows the aligned reads for gastrocnemius H3K4me3 **(b)**, H3K27ac **(d)** and H3K27me3 **(f)**. Males are in blue, females in pink.

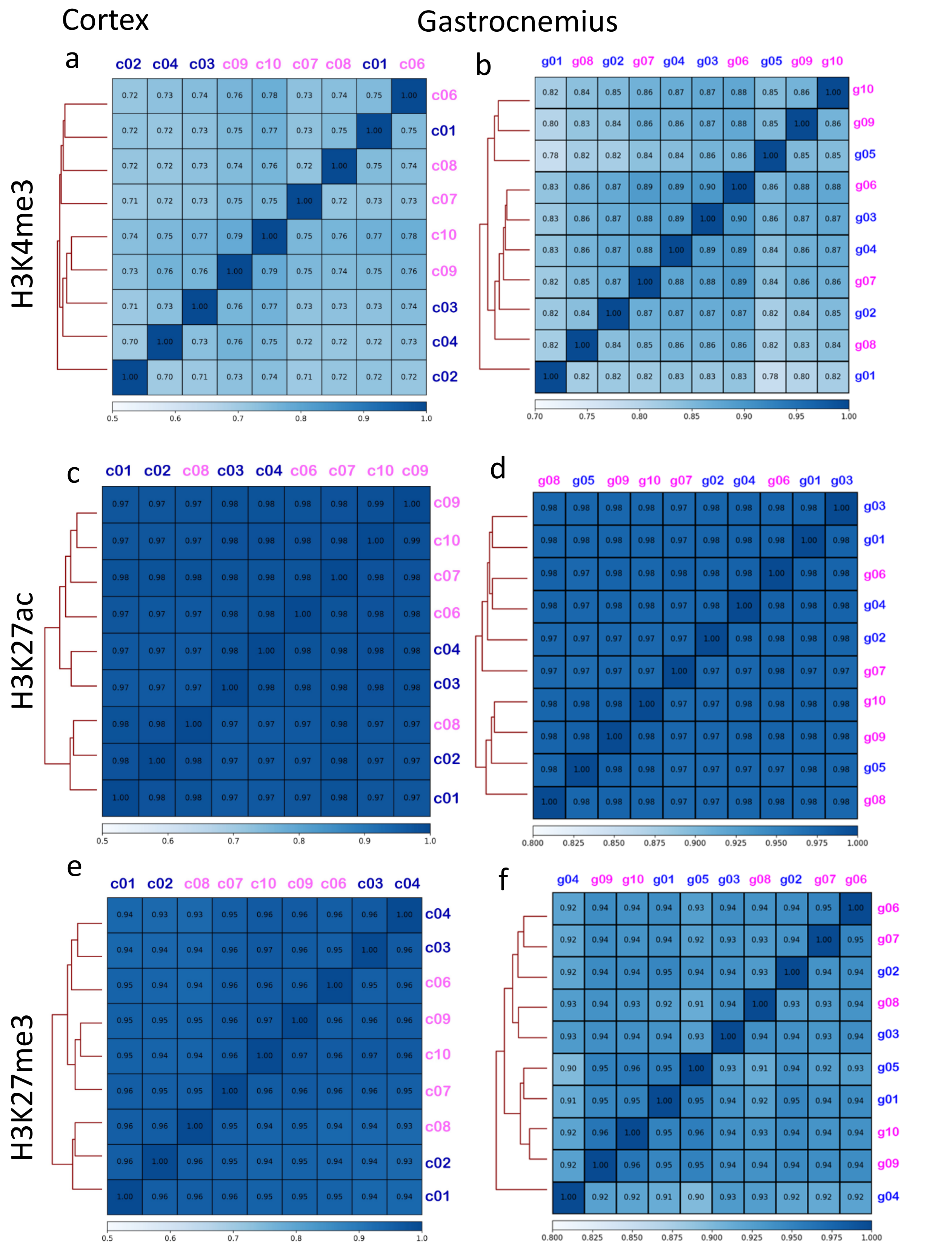

Figure S2. Spearman correlation including only autosomes. On the left side the Spearman correlation heatmap is represented including all the aligned autosomal chromosomes for H3K4me3 **(a)**, H3K27ac **(c)** and H3K27me3 **(e)** in brain. The corresponding graphs of gastrocnemius H3K4me3 **(b)**, H3K27ac **(d)** and H3K27me3 **(f)** are reported on the right part of the panel. Males are in blue, females in pink. The color of each cell represents the Spearman coefficient which ranges from 0.7 to 1 (highest correlation).

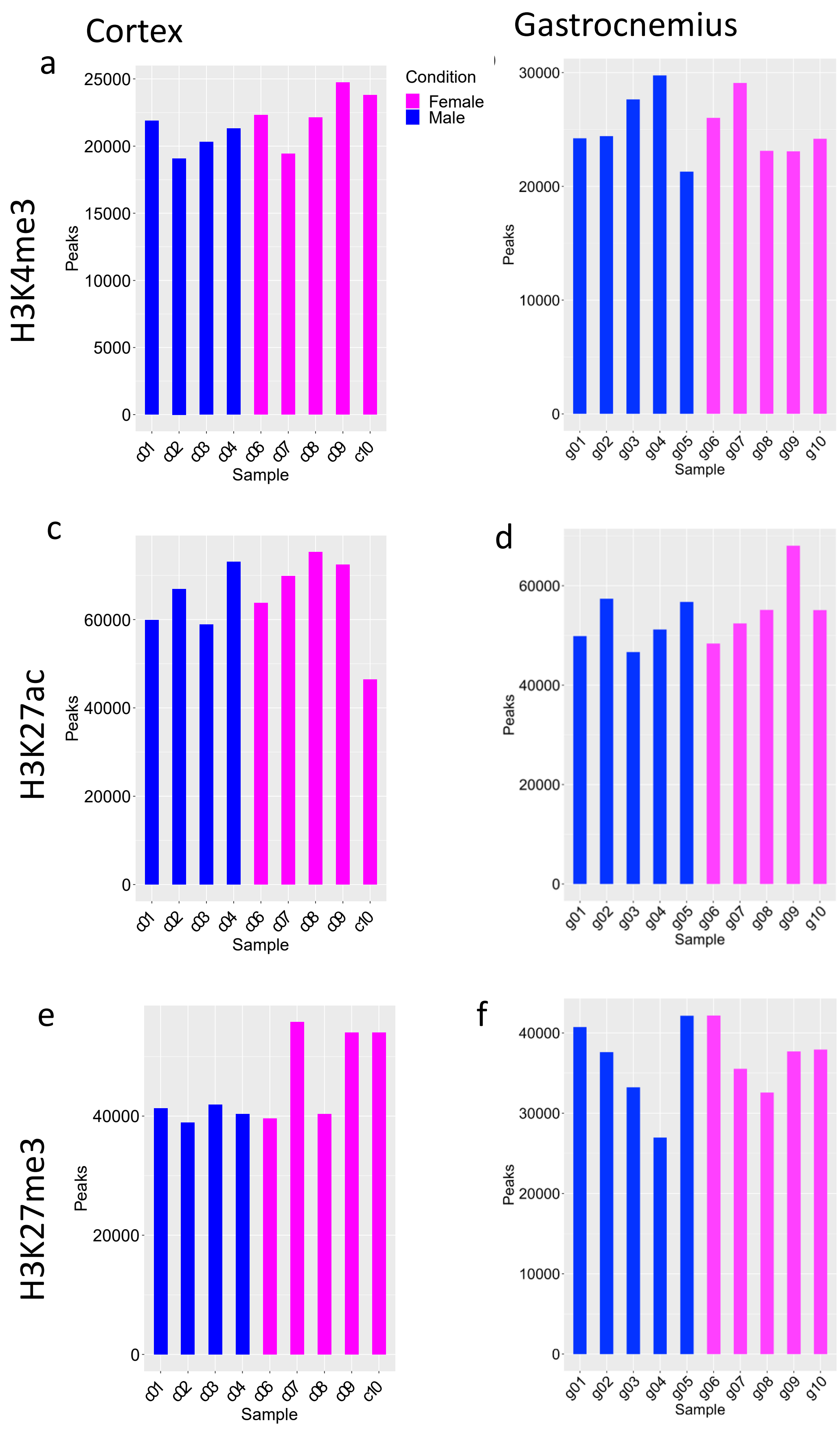

Figure S3. Number of peaks. On the left side the number of peaks for H3K4me3 **(a)**, H3K27ac **(c)** and H3K27me3 **(e)** in brain is reported. The right part of the figure shows the aligned reads for gastrocnemius H3K4me3 **(b)**, H3K27ac **(d)** and H3K27me3 **(f)**. Males are in blue, females in pink.

Table S1. Number of regions investigated during the differential bounding analyses. The number of regions investigated in each differential analysis is reported. Only peaks present in at least three samples of the considered group (females or males) were taken in consideration.

Histone modification	Chromosomes	N° of peaks Cortex	N° of peaks Gastrocnemius
H3K4me3	Autosomes	20962	22798
	X chromosome	655	629
H3K27ac	Autosomes	45236	31242
	X chromosome	738	719
H3K27me3	Autosomes	26482	18965
	X chromosome	4716	1283

Table S2. H3K4me3 differentially bound sites and related annotations in cortex. The number of the up and down regulated sites in females with respect to males is reported together with their feature distribution. Only sites with an FDR<0.1 were taken in consideration.

Chromosomes H3K4me3 (Cortex)	Feature	Up	Down
Autosomes	Promoters	0	1
	Gene Body	0	0
	Distal intergenic	0	1
	Total	0	2
X chromosome	Promoters	22	8
	Gene Body	14	2
	Distal intergenic	18	1
	Total	54	11

Table S3. H3K4me3 differentially bound sites and related annotations in gastrocnemius. The number of the up and down regulated sites in females with respect to males is reported together with their feature distribution. Only sites with an FDR<0.1 were taken in consideration.

Chromosomes H3K4me3 (Gastrocnemius)	Feature	Up	Down
Autosomes	Promoters	25	23
	Gene Body	15	35
	Distal intergenic	13	28
	Total	53	86
X chromosome	Promoters	40	10
	Gene Body	24	14

	Distal intergenic	22	2
	Total	86	16

Table S4. H3K27ac differentially bound sites and related annotations in cortex. The number of the up and down regulated sites in females with respect to males is reported together with their feature distribution. Only sites with an FDR<0.1 were taken in consideration.

Chromosomes H3K27ac (Cortex)	Feature	Up	Down
Autosomes	Promoters	0	1
	Gene Body	0	0
	Distal intergenic	1	0
	Total	1	1
X chromosome	Promoters	13	4
	Gene Body	24	4
	Distal intergenic	10	1
	Total	47	9

Table S5. H3K27ac differentially bound sites and related annotations in gastrocnemius. The number of the up and down regulated sites in females with respect to males is reported together with their feature distribution. Only sites with an FDR<0.1 were taken in consideration.

Chromosomes H3K27ac (Gastrocnemius)	Feature	Up	Down
Autosomes	Promoters	87	36
	Gene Body	170	157
	Distal intergenic	217	114
	Total	474	307
X chromosome	Promoters	13	4
	Gene Body	22	9
	Distal intergenic	14	7
	Total	49	20

Table S6. H3K27me3 differentially bound sites and related annotations in cortex. The number of the up and down regulated sites in females with respect to males is reported together with their feature distribution. Only sites with an FDR<0.1 were taken in consideration.

Chromosomes H3K27me3 (Cortex)	Feature	Up	Down
Autosomes	Promoters	0	0
	Gene Body	1	1
	Distal intergenic	3	0
	Total	1	1
X chromosome	Promoters	41	7
	Gene Body	61	10
	Distal intergenic	72	15
	Total	174	32

Table S7. H3K27me3 differentially bound sites and related annotations in gastrocnemius. The number of the up and down regulated sites in females with respect to males is reported together with their feature distribution. Only sites with an FDR<0.1 were taken in consideration.

Chromosomes H3K27me3 (Gastrocnemius)	Feature	Up	Down
Autosomes	Promoters	41	7
	Gene Body	61	10
	Distal intergenic	72	15
	Total	174	32
X chromosome	Promoters	95	152
	Gene Body	164	69
	Distal intergenic	155	69
	Total	414	220

Table S8. Biological signatures associated to H3K4me3-regulated promoters on cortex X chromosome. Ingenuity pathway analysis for promoters found to be differentially bound by H3K4me3 on brain X chromosome was carried out and results are reported here. Only terms with an activation z-score above |2| are taken in consideration. All the results are presented as regulated (increased/decreased) in females with respect to males.

Cortex Diseases or Functions (H3K4me3 X)	p-value	Predicted Activation State	Activation z-score	Molecules	N° of molecules
Frequency of tumor	1.74E-03	Decreased	-2.000	P1S2,CA5B,CDX4,DDX3X,DUSP9,EIF2S3,GPR34,HCCS, HUWE1,Jpx,KCND1,KDM5C,KDM6A,L1CAM,MAOB, MID1,PBDC1,PHKA2, RENBP,SLC38A5,TAB3,TLR7, UTP14A,XIST	24
Incidence of tumor	2.71E-03	Decreased	-2.000	AP1S2,CA5B,CDX4,DDX3X,DUSP9,EIF2S3,GPR34,HCCS, HUWE1,Jpx,KCND1,KDM5C,KDM6A,L1CAM,MAOB, MID1,PBDC1,PHKA2,RENBP,SLC38A5,TAB3,TLR7, UTP14A, XIST	24
Formation of solid tumor	7.12E-03	Decreased	-2.000	AP1S2,CA5B,CDX4,DDX3X,DUSP9,EIF2S3,GPR34,HCCS, HUWE1,Jpx,KCND1,KDM5C,KDM6A,L1CAM,MAOB, MID1, PBDC1,PHKA2,RENBP,SLC38A5,TAB3,TLR7, UTP14A,XIST, ZFP92	25

Table S9. Biological signatures associated to H3K4me3-regulated promoters on gastrocnemius X chromosome. Ingenuity pathway analysis for promoters found to be differentially bound by H3K4me3 on gastrocnemius X chromosome was carried out and results are reported here. Only terms with an activation z-score above |2| are taken in consideration. All the results are presented as regulated (increased/decreased) in females with respect to males.

Gastrocnemius Diseases or Functions (H3K4me3 X)	p-value	Predicted Activation State	Activation z-score	Molecules	N° of molecules
Development of digestive organ tumor	1.36E-05	Decreased	-2.000	ABCD1,ARHGAP36,CACNA1F,CXorf38,DACH2,DCAF 12L2, DDX3X,DUSP9,EIF2S3,GABRQ, GPR143,GPR50, HCCS,IGSF1, Jpx,KCND1, KDM5C,KDM6A,MAGEE2, MAGIX,MAOB,MID1, NAP1L3,PCDH11X,POU3F4, PTCHD1,SLITRK4, SOWAHD, SRPX,UTP14A,XIST	31
Development of carcinoma	4.00E-04	Decreased	-2.000	ABCD1,ARHGAP36,CACNA1F,CLCN5,CXorf38, DACH2,DCAF12L2,DDX3X,DUSP9,EIF2S3, GABRQ, GPR143,GPR50,HCCS,IGSF1,Jpx,KCND1, KDM5C, KDM6A,MAGEE2,MAGIX,MAOB,MID1,NAP1L3, PBDC1,PCDH11X,POU3F4,PTCHD1, RAB9B,SLITRK4, SOWAHD,SRPX,UTP14A,XIST	34

Table S10. Biological signatures associated to H3K27ac-regulated promoters on gastrocnemius autosomes**.** Ingenuity pathway analysis for promoters found to be differentially bound by H3K27ac on gastrocnemius autosomal chromosomes was carried out and results are reported here. Only terms with an activation z-score above |2| are taken in consideration. All the results are presented as regulated (increased/decreased) in females with respect to males.

Gastrocnemius Diseases or Functions (H3K27ac autosomes)	p-value	Predicted Activation State	Activation z-score	Molecules	N° of molecules
Rheumatic Disease	1.71E-04	Increased	2.117	ALOX5AP,BDNF,FGFR3,GFI1,HNMT,IFI44L,IL15,IRS1, LRTM1,Masp1,mir-1,PGR,PRUNE2,PTPN1,SLC7A2, SYNE1, TMEM178A,TMEM266,TNFRSF11A,VTCN1	20
Inflammation of joint	3.73E-04	Increased	2.117	ALOX5AP,BDNF,FGFR3,HNMT,IL15,IRS1,Masp1,PGR, PRUNE2,PTPN1,SLC7A2,SYNE1,TMEM178A, TMEM266, TNFRSF11A,VTCN1	16
Proliferation of epithelial cells	1.18E-03	Increased	2.396	BDNF,FGFR3,IL15,INHA,mir-204,PGR,PTPN1, SEMA3C,TNFRSF11A,TRIM24	10
Growth of epithelial tissue	1.52E-03	Increased	2.683	ANGPT1,BDNF,FGFR3,IL15,INHA,ITGA4,mir-204,PGR, PTPN1,SEMA3C,TNFRSF11A,TRIM24	12
Apoptosis	2.97E-03	Decreased	-2.096	ACTC1,ANGPT1,BDNF,BMP8A,CA3,CES1,FGFR3,GFI1, IL15, INHA,IRS1,ITGA4,mir-1,mir-204,MUSK,MYCN, PGR,PKNOX2, PRDM9,PTPN1,RNF13,SEMA3C,SOCS2, SUB1,SYNE1, TNFRSF11A,TRIM24,TSC22D1,VTCN1	29

Table S11. Biological signatures associated to H3K27ac-regulated promoters on gastrocnemius X chromosome. Ingenuity pathway analysis for promoters found to be differentially bound by H3K27acon brain X chromosome was carried out and results are reported here. Only terms with an activation z-score above |2| are taken in consideration. All the results are presented as regulated (increased/decreased) in females with respect to males.

Gastrocnemius Diseases or Functions (H3K27ac X)	p-value	Predicted Activation State	Activation z-score	Molecules	N° of molecules
Frequency of tumor	2.92E-02	Decreased	-2.000	DDX3X,EIF2S3,GPR34,HCCS,Jpx,KDM5C,KDM6A, MID1,PBDC1,PLS3,UTP14A,XIST	12
Incidence of tumor	3.66E-02	Decreased	-2.000	DDX3X,EIF2S3,GPR34,HCCS,Jpx,KDM5C,KDM6A, MID1, PBDC1,PLS3,UTP14A,XIST	12

Table S12. Diet composition.

Ingredients	g/kg diet
**Protein, of which**	**112.5**
casein	*111*
DL-methionine	*1.5*
**Carbohydrate, of which**	**722**
corn starch	*490*
maltodextrin	*132*
sucrose	*100*
**Fat, of which**	**70**
soybean oil	*70*
**Other Additives**	**95.414**
cellulose	*48*
Tert-butylhydroquinone	*0.014*
mineral mix (TD94046)	*35*
vitamin mix (CA40060)	*10*
potassium phosphate monobasic	*2.4*
